# Intratarsal Keratinous Cyst Misdiagnosed as a Chalazion Complicated by an Intralesional Triamcinolone Injection: A Case Report

**DOI:** 10.7759/cureus.109075

**Published:** 2026-05-18

**Authors:** Ken-Ichi Takaki, Mika Tanabe, Hiroshi Yoshikawa, Koh-Hei Sonoda

**Affiliations:** 1 Ophthalmology, Takaki Eye Clinic, Saga, JPN; 2 Ophthalmology, Kyushu University, Fukuoka, JPN

**Keywords:** chalazion, eyelid inflammation, intralesional injection, intratarsal keratinous cyst, triamcinolone acetonide

## Abstract

An intralesional triamcinolone acetonide injection is one of the treatment options for chalazia. However, lesions clinically resembling chalazia may occasionally represent other pathological entities, potentially leading to unexpected complications after treatment.

We report the case of a 54-year-old man with a left upper eyelid mass initially diagnosed as a chalazion and treated with an intralesional triamcinolone acetonide injection. The following day, the lesion showed marked worsening, with swelling, redness, and pain. Imaging revealed a cystic lesion, and bacterial infection could not be excluded. Intravenous antibiotics were initiated; however, the lesion continued to worsen, and drainage yielded sterile purulent material.

Surgical excision revealed a cystic lesion containing keratinous material. Histopathological examination demonstrated marked inflammatory cell infiltration without granulomatous inflammation or lipid-laden macrophages characteristic of chalazion. Based on the surgical findings, histopathological findings, and clinical course, the lesion was diagnosed as an intratarsal keratinous cyst complicated by rupture and foreign body inflammatory reaction following intralesional steroid injection.

This case highlights the importance of careful differential diagnosis in eyelid lesions mimicking chalazia. An intralesional steroid injection may cause severe inflammatory complications when administered to inappropriate lesions.

## Introduction

An intralesional triamcinolone acetonide injection is a standard treatment option for a chalazion, along with warm compresses and incision and curettage, and it is considered safe and effective therapy [1]. Previous reports have described complications such as vision loss due to vascular occlusion following an intralesional triamcinolone acetonide injection [2]. However, eyelid-related complications are limited to fat atrophy and skin depigmentation [3], and severe conditions, such as acute eyelid inflammation, have rarely been reported.

However, when this treatment is applied to lesions that mimic chalazia but represent different diseases, it may result in inappropriate management. An intratarsal keratinous cyst is a rare eyelid lesion that may clinically mimic a chalazion [4,5].

We herein report the case of an intratarsal keratinous cyst that was misdiagnosed as a chalazion and thus was treated with an intralesional triamcinolone acetonide injection, which resulted in acute eyelid inflammation due to a foreign body reaction.

## Case presentation

A 54-year-old man presented with a left eyelid mass with an approximate 1-month duration. At a local clinic, the lesion was initially diagnosed as a chalazion, and an intralesional triamcinolone acetonide injection was performed. According to the referral information, triamcinolone acetonide was injected directly into the lesion; however, the exact dose and injection technique were unavailable. The following day, the swelling of the lesion worsened, and it was accompanied by redness and pain.

Two days after the onset of symptoms, the patient was referred to our department. At presentation, his best-corrected visual acuity was 20/20 in both eyes. The intraocular pressure was 10 mmHg in the right eye and 18 mmHg in the left eye. Marked edema and erythema of the left upper eyelid were observed, with difficulty in opening the eyelid (Figure 1A).

**Figure 1:**
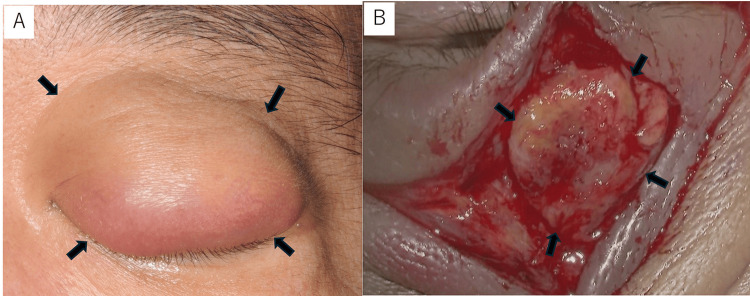
Clinical and intraoperative findings (A) Marked swelling and erythema of the left upper eyelid at presentation, with difficulty in eyelid opening. Black arrows indicate the swollen upper eyelid. (B) Intraoperative surgeon’s view showing a yellow-white lesion beneath the skin, continuous with a cystic structure. Black arrows indicate the cystic lesion.

Contrast-enhanced computed tomography revealed a well-defined, uniformly cystic lesion measuring 37 × 24 × 10 mm in the left upper eyelid, with cyst wall enhancement (Figures 2A, 2B). No internal septations were observed.

**Figure 2:**
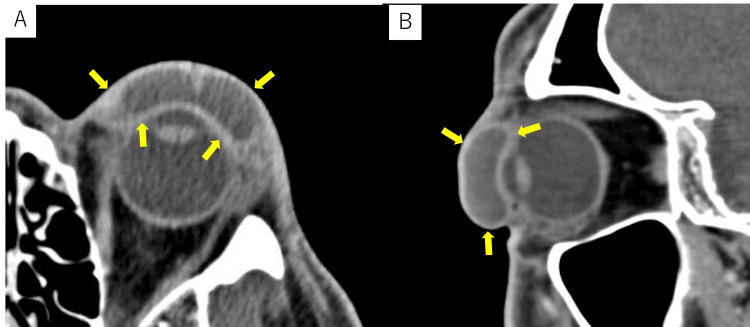
Imaging findings (A) Axial contrast-enhanced computed tomography demonstrating a well-defined cystic lesion in the left upper eyelid, with enhancement of the cyst wall. No internal septations are observed. Yellow arrows indicate the cystic lesion. (B) Sagittal contrast-enhanced computed tomography demonstrating a well-defined cystic lesion in the left upper eyelid, with enhancement of the cyst wall. No internal septations are observed. Yellow arrows indicate the cystic lesion.

Because differentiation from a bacterial infection was difficult and the possibility of a severe bacterial infection was considered, intravenous ceftriaxone (2 g/day) was administered. However, as the eyelid swelling continued to worsen, needle aspiration and drainage were performed on day 3 after onset. A yellowish purulent fluid was thus obtained. Bacterial cultures of the aspirated material showed no growth. After drainage, the eyelid swelling gradually improved, and no re-exacerbation was noted.

On day 8 after onset, the mass was surgically excised using a transcutaneous approach. A yellowish-white lesion was observed beneath the skin, continuous with a cystic structure (Figure 1B). The cyst protruded from the tarsal plate in a pedunculated manner, with minimal adhesion to the surrounding tissues. Keratinous material leaked from the cyst, and no obvious atheromatous material was observed either within or around the lesion.

A histopathological examination revealed a cystic lesion containing keratinous material (Figures 3A, 3B) with marked inflammatory cell infiltration in the cyst wall (Figure 3B). No epithelial lining was identified, possibly due to inflammatory destruction. Macrophages containing lipid droplets, a characteristic of chalazia, were not observed. These findings were clearly different from the granulomatous inflammation typically seen in chalazia.

**Figure 3:**
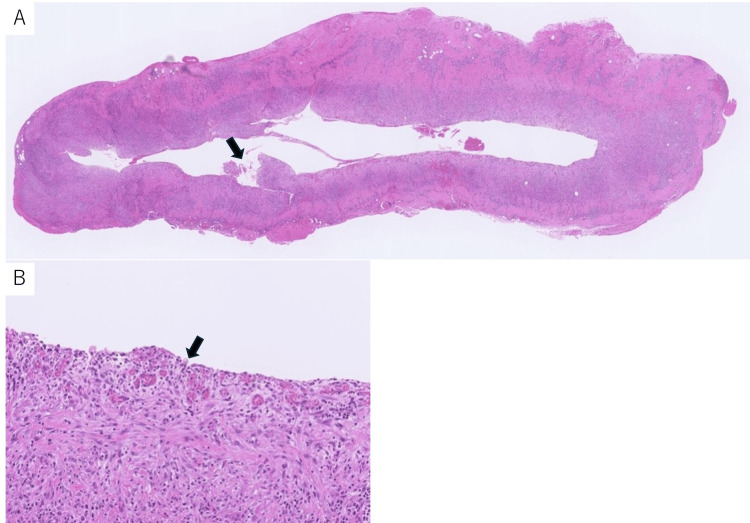
Histopathological findings (A) Low-power histopathological view showing a cystic structure containing keratinous material. The black arrow indicates the keratinous material. (B) High-power histopathological view demonstrating marked inflammatory cell infiltration in the cyst wall. The black arrow indicates the inflamed cyst wall. No epithelial lining is clearly identified, and no lipid-laden macrophages characteristic of a chalazion are observed.

Based on the intraoperative findings, histopathology, and clinical course, a rupture of an intratarsal keratinous cyst induced by an intralesional triamcinolone injection was suspected, leading to keratin leakage and a subsequent foreign body inflammatory reaction. A diagnosis of an intratarsal keratinous cyst was made based on the clinical, intraoperative, and histopathological findings.

Given the non-infectious nature of the inflammation, ceftriaxone was discontinued on day 10 after confirming the negative culture results. The patient’s eyelid swelling improved rapidly after tumor excision, and no recurrence was observed during the six-month follow-up period.

## Discussion

We report a case of acute eyelid inflammation following an intralesional triamcinolone acetonide injection.

It is well-known that the rupture of an epidermal cyst can induce a marked inflammatory response due to the release of keratin into the surrounding tissues [6]. In the present case, an intralesional steroid injection may have increased the intracystic pressure, resulting in disruption of the cyst wall and leakage of keratinous material into the surrounding tissue, thereby triggering a foreign-body inflammatory reaction. In addition, negative bacterial cultures and the rapid improvement observed after drainage and surgical excision further support a non-infectious etiology.

Intratarsal keratinous cysts are rare cystic lesions thought to originate from squamous metaplasia and cystic dilatation of the meibomian gland duct within the tarsal plate [4]. They are often misdiagnosed as chalazia because of their similar clinical appearance. Although detailed pre-treatment clinical findings were unavailable because the patient was initially treated at another clinic, previous reports have described several clinical characteristics that may retrospectively distinguish intratarsal keratinous cysts from chalazia. Intratarsal keratinous cysts are typically slowly enlarging lesions located away from the eyelid margin and are often associated with relatively limited surrounding inflammation compared with chalazia [4,5]. In addition, recurrent, atypical, or unusually firm eyelid lesions should prompt consideration of alternative diagnoses, including intratarsal keratinous cysts and eyelid tumors. Furthermore, eyelid tumors such as sebaceous carcinoma have sometimes been initially misdiagnosed as chalazia [7], highlighting the importance of a careful differential diagnosis when evaluating eyelid masses resembling chalazia.

In contrast to secondary epidermal inclusion cysts, which are typically associated with prior trauma or surgery and arise from the implantation of epidermal elements into the dermis or subcutaneous tissue, intratarsal keratinous cysts characteristically arise within the tarsus and show continuity with the tarsal plate. In the present case, no history of prior eyelid trauma or surgery was identified, and the intraoperative findings demonstrated continuity between the cyst and the tarsal plate, supporting the diagnosis of an intratarsal keratinous cyst. Although the epithelial lining was not preserved histologically, likely because of inflammatory destruction after cyst rupture, the diagnosis of intratarsal keratinous cyst was favored in the present case based on the characteristic intraoperative findings and lesion location. Intraoperatively, the cyst showed continuity with the tarsal plate and protruded in a pedunculated manner from the tarsus, findings that were more consistent with an intratarsal keratinous cyst than with an epidermal inclusion cyst or epidermoid cyst, which typically arise from the skin or subcutaneous tissue.

In the present case, marked acute inflammation developed rapidly after the intralesional steroid injection, possibly due to cyst rupture and keratin leakage. Although direct histopathological evidence of complete cyst wall rupture was not identified, intraoperative leakage of keratinous material from the cyst suggested focal disruption of the cyst wall. In addition, the abrupt onset of marked inflammation immediately after intralesional steroid injection supports the possibility that increased intracystic pressure contributed to keratin leakage and subsequent foreign-body inflammation. Alternative explanations, including a sterile inflammatory reaction induced by the injection itself, cannot be entirely excluded.

Although imaging findings of intratarsal keratinous cysts have not been well-established, the present lesion demonstrated a well-defined homogeneous cystic structure without internal septations or invasive features on contrast-enhanced computed tomography. These findings were more consistent with a benign cystic lesion than with an aggressive neoplastic process. However, because marked inflammatory changes were present clinically, differentiation from chalazion or abscess based on imaging alone remained difficult. Therefore, a definitive diagnosis required correlation with intraoperative and histopathological findings.

An intralesional triamcinolone acetonide injection has been reported to have treatment outcomes comparable to incision and curettage for chalazion and is considered an effective treatment option [1]. However, if an intratarsal keratinous cyst is misdiagnosed as a chalazion, intralesional triamcinolone injection may lead to severe eyelid inflammation due to a foreign-body reaction. Therefore, careful differential diagnosis is essential prior to treatment initiation.

## Conclusions

This case highlights the potential risk of administering intralesional steroid injections without sufficient diagnostic consideration in eyelid masses presumed to be chalazia. In cases with atypical features, re-evaluation of the diagnosis and careful determination of the treatment strategy are warranted.
